# Abstracts from Foreign Journals

**Published:** 1902-01

**Authors:** 


### ABSTRACTS FROM FOREIGN JOURNALS.

### FRENCH.

UNDER THE DIRECTION OF M. P. RAVENEL, M.D., AND S. J. J. HARGER, V.M.D., OF PHILADELPHIA, PA.

TUBERCULOSIS IN THE HORSE. Markees asserts, with Nocard, that in the abdominal form of tuberculosis polyuria is a diagnostic symptom of the first order; also that this form of the disease is of avian origin.—*Bull. Vét.*

BENZINE AS AN ANTHELMINTIC. Graal recommends, instead of the usual anthelmintics, benzine in doses of 10 to 30 grammes. —*Ibid.*

RAW MILK IN MARASMUS AND INTESTINAL CATARRH OF THE NEWBORN. Boiled or sterilized milk undergoes during this process certain alterations : the casein becomes uncoagulable, the nuclein and lecithin insoluble, the lactose modified, and the fat collects in droplets upon the surface. Jensen and von Starck have observed young calves fed upon boiled milk to emaciate and die. Rhachitism is attributed to it by Heubner. Monrad has noticed that in marasmus (athrepsia) and intestinal catarrh of the newborn Pasteurized milk is not assimilated. The danger from crude milk consists in tuberculosis and the coli bacillus. The former can be prevented by proper sanitary laws and the latter by cleanliness on the dairy farm. Monrad does not advise the suppression of sterilized milk, but opposes its use in marasmus and intestinal catarrh.—*Bull. Vét.*

DERMOID CYST OF THE GREAT OMENTUM. Petit, of Alfort, found a dermoid cyst in the great omentum of a horse that had died from tetanus. The tumor, which was flat and discoid in shape, measured 50 cm. in diameter. It was hollowed by several cavities as large as a walnut, which contained a thick, mortar-like substance containing hairs 20 to 30 cm. long and agglutinated by sebum.—*Rev. Vét.*

TUBERCULOSIS OF THE HUMERUS. Cadeac and Morot found in the lungs and lymphatic glands the lesions of tuberculosis of the upper end of the humerus. The latter was enlarged to three times its normal size, and on section showed numerous cavities filled with the tuberculous matter.—*Rev. Vét.*

Transmission of Warts from the Heifer to Man. Bédel, in removing several warts from the udder of a heifer, accidentally cut his left index finger. About three or four months afterward three papillomas as large as a grain of wheat developed at this point. With Dr. Majocchi, who gave a similar instance, he incriminates contagion. Bédel also observed a papillomatous tumor, as large as a hazel-nut, in the inferior lips of a foal at birth; the dam carried numerous warts on the lips and nostrils. Bédel therefore believes that warts are congenital and hereditary.—*Rev. Vét.*

Suturing and Resection of the Trachea. Mesnard made such experiments upon the dog. Two, three, and five rings were removed in different dogs, and the two ends of the trachea drawn together and sutured with catgut. In ten days the cicatrization was complete; the animals retained their normal state. Mesnard also resected six rings from the trachea of one dog and transplanted them into that of another dog, and *vice versa*. The result was complete.

The author suggests the application of these operations in the larger animals, especially the horse, in which there may be limited narrowing or obstruction of this tube from diverse causes.—*Rev. Vét.*

Piroplasma Canina—Tick Disease in the Dog. Almy has observed at Alfort five new cases of piroplasma canina or tick disease, which seems to affect principally hunting dogs. In all cases the skin was covered with ticks, and the red blood cells contained the *piroplasma bigeminum*. The principal symptoms were stupidness, inappetence, fever (sometimes 40° C.), hæmoglobinuria; in some cases an icteric tint of the mucosa and skin; red blood cells were always diminished—in one case 1,260,000 per c.c. Three out of five animals died; those surviving were anæmic for a long time. Subcutaneous injections of hydrobromate of quinine were not very successful.

Leblanc thinks that the tick theory is exaggerated, and associates the disease with the African disease tristeza.

Nocard observed ticks in all cases, but the presence of ticks is not always positive of the disease, because some species of ixodes are non-pathogenic.

A dog may become immune through the repeated bites of a small number of ticks. Experiment has shown that the injection of a small quantity of infected blood into a healthy dog makes him immune to a large dose of such blood ordinarily fatal when injected alone.—*Rev. Vét.*

THERAPEUTIC REVIEW. *Œdema.* Against œdema and tumors of the members consecutive to traumatism Colin employs a mixture of carbonate of calcium, 100; sulphate of iron, 30 ; and acetic acid, 9.5 parts. For subcutaneous œdema and hydropsy of cavities Janzon employs potassium iodide.

*Mange.* Müller and Regenbogen conclude that *epicarin* has no more merit than other remedies used for *sarcoptic mange in the dog.* Frick, Brandi, and Gmeiner, who recommend epicarin, also employed with success saponified cresyl. The following liniment is recommended : Cresolinated water (cresol-water 9), 500; potash soap, 250; alcohol, 250 parts, applied with strong friction every two days Five applications suffice.

Against *demodectic mange* in the dog Poth shaves the epidermis before applying the remedy (previously reported). Paust has cured two cases as follows : Thorough washing with soap and hot water, morning and evening, and then with a solution of creolin ; then wash for several minutes with a strong decoction of tobacco, dry the skin, and apply the following ointment : Sulph. sublim., 1; zinc. sulph., 1; axony, 9 parts.

Lemke gives preference to an alcoholic solution of corrosive sublimate, 0.5 to 1.25 per cent. The sublimate may also be incorporated in vaseline. In thin-skinned dogs 0.5 per cent. suffices. He also suggests iodine, chloroform, and oil of turpentine, 1 part each, in liquid vaseline, 2 parts. Vogg advises the following : Ointment of zymoidine of Rosenberg, 10 ; purified lanolin, 60; yellow vaseline, 10 parts. The hair is cut off, the skin washed with soap and warm water, and three weekly applications made. Wagenaar washes the mangy spots with pure creolin until the skin is inflamed, which is then washed twice daily with an 8 per cent. solution of creolin.

Mathis has treated *mange in the sheep* with essence of lavender or essence of turpentine.

Mange in the horse is treated with a liniment composed of lysol, tar, soap, and alcohol. On the extremities the following formulæ can be used : Lysol, 5 per cent. in water; benzine mixed in three times its weight of linseed oil ; naphthalin ointment.

Imminger prescribes the following: Sublimed sulphur, 50 g.; mercurial ointment, 50 g.; powdered cantharides, 20 g.; these are carefully mixed and added to 400 g. of vaseline. This is applied to the diseased parts, which in two days are washed with soap and warm water.

*Trichorrhexis* (*falling out of the hair*). Kutzner and Reichert had good results in trichorrhexis of the tail in the horse with a 1 per cent. solution of pyoctanin blue (yellow in sorrel horses). The tail is first washed with a 3 per cent. alkaline solution. Goldbeck recommends daily washing with a 5 per cent. solution of pyrogallol.—*Rev. Vét.*

CHROMIC ACID IN APHTHOUS FEVER. Carani treated a large number of cases of aphthous fever with a concentrated solution of chromic acid, 30 per cent. Two applications daily for two days suffice to complete the cicatrization of the ulcers of the interdigital space.—*Bull. Vét.*

JABOT OF THE JUGULAR VEIN IN THE HORSE. The case presented to Garibaldi had for several days been unable to drink. Food and water were ejected through the nostrils, and what was swallowed was arrested at the superior third of the neck along the course of the jugular vein, forming a fusiform swelling 12 cm. long and 6 cm. wide. Dilatation of the œsophagus was eliminated: (1) When the jugular was compressed below the tumor returned; (2) puncture of the swelling with a Provaz needle showed it to contain blood.

The treatment consisted in dissecting the dilated jugular and ligation above and below the dilatation. In two days the animal was able to swallow.—*Bull. Vét.*

---

### GERMAN.

THE USE OF ASSES' MILK FOR INFANTS. In the *Munich mediz. Wochenschrift*, No. 45, 1901, a report is given of a lecture on the above subject by Dr. R. Klemm. It was claimed that asses' milk is more free from microbes and toxins than cows' milk, and that it is more nourishing, digestible, and palatable than cows' milk. It is to be preferred both for sick and for well children. One farm supplied 9500 marks' worth of this food during 1900, and it was prescribed by sixty physicians.

Glanders in Alsace. There has been so much glanders in Alsace that a police order has been issued to the following effect: Shippers bringing horses into the province are required to advise the police by telegraph where and when the horses will be first stabled. The horses are examined by the district veterinarian and are placed under quarantine. During this quarantine the horses may be worked, but if they are sent to another location or are sold the police must be notified. Every three weeks the horses are re-examined, and reports are made to the bureau of police. At the end of three months, if the horses are healthy, the quarantine is discharged. The cost of these inspections is placed on the owner. —*Deutsch. med. Wochenschrift*, January 4, 1902.

German Meat-inspection in China. In the *Med. Wochenschrift*, No. 45, 1901, the following information is given from the letter of a German veterinarian in China. Of 287 Chinese swine 32 had distomata in the liver, 22 had trichinosis, 3 had erysipelas (rothlauf), 1 had echinococci, and 9 had cysticercus cellulose; 110 goats and sheep were sound; of 34 Chinese cattle 3 had cysticercus inermis, 1 a pulmonary abscess, 1 tuberculosis; of 18 calves 2 had pneumonia. Among fowls many were found with a unilateral inflammation of the eye, in which the eyeball broke down into a yellow, sticky mass. It was found that this product contained the chicken cholera bacillus in pure culture. Engel has strongly recommended the use of balsam of Peru mixed with an equal quantity of alcohol as an injection in cartilaginous quittor.

Behring's Recent Announcement in Regard to Tuberculosis of Cattle. Professor Behring, the discoverer of diphtheria antitoxin, has been awarded the Nobel prize for making the most important recent contribution to knowledge in the field of medicine. Upon the occasion of the awarding of the prize ($12,000), in the Academy of Science in Stockholm, Professor Behring announced some of the more recent results of his investigations in regard to tuberculosis. Behring has been experimenting with cattle in the production of immunity to tuberculosis by inoculating them with a non-virulent culture of tubercle bacilli. He holds that when this is done an immunity is acquired that will enable the animal to resist virulent tubercle bacilli. As a parallel he calls attention to the protective effects of anthrax vaccine, wherein a weakened culture is inoculated into an animal to enable it later to resist a virulent culture. He announced his intention to use the prize fund to pay for further experiments along this line, and expressed a strong hope that a method of immunizing cattle against tuberculosis on a practical scale would eventually be found. He also said that he had found human tuberculosis virulent for cattle, and believed both to be minor variations of the same disease.

It is to be hoped that this expectation will be realized. The State Live-stock Sanitary Board of Pennsylvania has also been working on this problem for many months, and, perhaps, is approaching the same result.

